# Pharmacogenetic Study of the Impact of *ABCB1* Single Nucleotide Polymorphisms on the Response to Cyclosporine in Psoriasis Patients

**DOI:** 10.3390/pharmaceutics14112441

**Published:** 2022-11-11

**Authors:** Alexandr Chernov, Daria Kilina, Tatiana Smirnova, Elvira Galimova

**Affiliations:** 1 Department of Life Sciences, Ben-Gurion University, Beer Sheva 84105, Israel; 2 Department of General Pathology and Pathological Physiology, Institute of Experimental Medicine, St. Petersburg 197376, Russia; 3 HLA Laboratory, V.A. Almazov National Medical Research Center of the Ministry of Health of Russia, St. Petersburg 197341, Russia; 4 City Dispensary of Dermatology and Venereology, St. Petersburg 192102, Russia; 5 Interdisciplinary Laboratory for Neurobiology, Sechenov Institute of Evolutionary Physiology and Biochemistry, Russian Academy of Sciences, St. Petersburg 194223, Russia

**Keywords:** pharmacogenetics, psoriasis, cyclosporine, unresponsiveness, ABCB1 gene, polymorphisms, population, personalized therapy

## Abstract

Psoriasis is a chronic, T cell-mediated skin disease affecting 2–3% of the Caucasian population. Cyclosporine A is a calcineurin inhibitor that acts selectively on T cells. The cyclosporine A treatment response has been suggested to be modulated by single-nucleotide polymorphisms (SNPs) in the *ABCB1* gene. The aim of this research was to evaluate the effect of *ABCB1* genetic variants that could affect the response to a cyclosporine treatment in Russian psoriasis patients with the *ABCB1* genotype status. The *ABCB1* T-129C, G1199A, C1236T, G2677T/A and C3435T SNPs in the 168 patients with psoriasis were genotyped by PCR-RFLP (polymerase chain reaction-restriction fragment length polymorphism) and TaqMan SNP genotyping assays. The *ABCB1* C1236T, G2677T/A and C3435T SNPs were significantly associated with a negative response to cyclosporine therapy. A very strong association was evident for the C3435T SNP in the *ABCB1* gene in the allele, dominant and recessive models (OR = 2.58, OR = 4.01, OR = 2.50, respectively). *ABCB1* C1236T and G2677T/A polymorphisms were significantly associated with a negative response to the cyclosporine therapy in the codominant, dominant and recessive models (*p* ˂ 0.05). Additionally, the haplotype analysis identified that the TGC haplotype is significantly associated with a negative response to cyclosporine therapy in psoriasis patients (*p* ˂ 0.05). The current study to the best of our knowledge is the first of its kind to be performed in the Russian population. In conclusion, the present results suggest an association between the *ABCB1* genetic variants and unresponsiveness to cyclosporine in the Russian population. Further, larger studies are necessary to confirm our findings and replicate them in various ethnic populations before its implementation in the clinical practice.

## 1. Introduction

**Figure 1:**
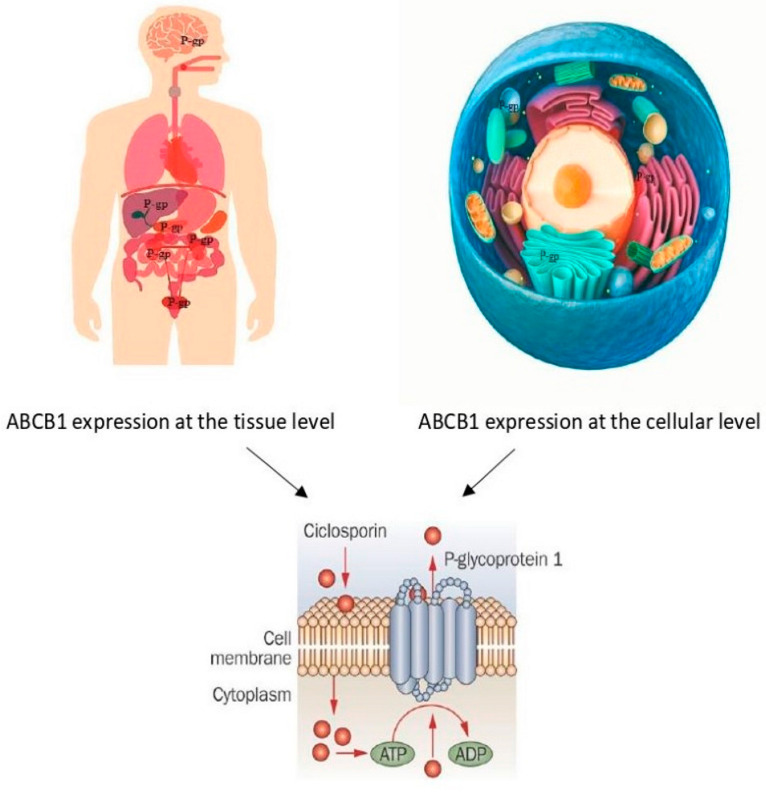
PGP (P-gp P-glycoprotein, ABCB1 (ATP-binding cassette sub-family B member 1), MDR1 (multidrug resistance protein 1), and CD243 expression in organs with excretory roles (e.g., liver, kidney and small intestine) and at blood–tissue barriers (e.g., blood–brain, blood–testis and blood–placenta), plasma membrane, Golgi apparatus, endoplasmic reticulum, lysosomes and endosomes and cyclosporine transport.

Psoriasis is a chronic T cell-mediated skin disease affecting 2–3% of the Caucasian population. Cyclosporine A (CsA) is a calcineurin inhibitor that acts selectively on T cells, thereby affecting the production of multiple inflammatory cytokines. CsA is used for the treatment of severe psoriasis, showing significant variability in its efficacy, which is associated with varying degrees of toxicity [1]. In particular, CsA is associated with several side effects including neurotoxicity, hypertension, hyperlipidemia and chronic nephrotoxicity. The absorption of CsA is highly variable in psoriasis patients which can be explained by the function and expression of the multidrug efflux transporter P-gp P-glycoprotein (MDR1 Multidrug Resistance Protein 1, ABCB1 ATP Binding Cassette Subfamily B Member 1) (Figure 1).

P-gp performs the function of the ATP-dependent efflux transporter pump for the substrates from the cytoplasm to the extracellular space [2]. ABCB1 plays a key role in the occurrence of adverse events and the efficacy of the treatment with CsA, methotrexate, etanercept, topical steroids, biologic agents and other [3]. At the tissue level, ABCB1 is expressed in the brain, skin, liver, pancreas, kidney, intestine, testis, ovary, endometrium, placenta and adrenal glands as well as in the blood–brain and blood–placenta barriers (Figure 1) [4]. At the cellular level, the expression of ABCB1 was established in the plasma membrane, Golgi apparatus, endoplasmic reticulum, lysosomes and endosomes [5,6]. The expression of ABCB1 in psoriasis lesional skin was detected to be significantly decreased [7]. ABCB1 expression was inhibited by CsA in the peripheral blood monocytes of rheumatoid arthritis patients [8]. Lown K.S. et al. revealed the significant role of intestinal P-glycoprotein in the first-pass elimination of CsA [9]. In mdr1a (murine MDR1 homologue) knockout mice, which were compared with wild-type mice, the CsA levels in the brain were significantly higher after the intravenous cyclosporine administration [10].

The multidrug resistance 1 (*MDR1* or *ABCB1*) gene, which encodes a 170-kDa P-glycoprotein, is found on chromosome 7 and contains 29 exons. *ABCB1* is a highly polymorphic gene. More than 990 *ABCB1* SNPs have been identified, the allelic frequencies of which varies widely among different populations [11]. In general, it has been demonstrated that *ABCB1* SNPs are detected in all of the studied populations [11,12]. Among the *ABCB1* SNPs, three polymorphic variants, C3435T in exon 26, C1236T in exon 12 and G2677T/A in exon 21, were extensively investigated and were identified functionally important. African American people (10%) have the lowest frequencies of polymorphic alleles when they were compared with Caucasian people (42–46%) and Asian people (45%). Tang K. et al. examined the haplotypes and linkage disequilibrium architecture of the *ABCB1* gene in Caucasian, African American, Chinese, Malaysian and Indian populations [13]. This study reported that the major haplotype 1236T-2677T-3435T is highly represented in the non-African populations, while the haplotype 1236C-2677G-3435C in the African American population indicating a varied haplotype diversity between the different populations. Kimchi-Sarfaty et al. performed C1236T, G2677T/A and C3435T SNPs analyses, and they determined their haplotypes in the Ashkenazi Jewish population [12]. The Ashkenazi Jewish population is similar to the Caucasian population in the allele and genotype frequencies, and the frequency of the common 1236T-2677T-3435T haplotype is 23.6% [12]. In another study, the frequency of the 3435C allele was lower in near Eastern Jewish population (0.445) and the Mediterranean group (0.58), while the frequencies of the C allele were quite comparable among the Ashkenazi (0.65), Yemenite (0.645), and North-African (0.615) Jewish populations [14].

Pharmacogenetic and pharmacogenomics studies have demonstrated the influence of the polymorphisms of drug-metabolizing enzymes, transporters and receptors in variable drug responses. Nevertheless, pharmacogenetic investigations of CsA are scarce. Single nucleotide polymorphisms (SNPs)/mutations and epigenetics modifications affect the expression and function of [15,16,17,18] P-gp, contributing to inter-individual and inter-ethical variabilities in the drug response and susceptibility to diseases. The SNPs have also been associated with several toxic effects [15]. Wang R. investigated the effect of common *ABCB1* C1236T, G2677T/A and C3435T genetics variants on the sensitivity, intracellular accumulation, and efflux of tacrolimus, cyclosporine A, sirolimus and everolimus in transfected LLC-PK1 cells [19]. ABCB1 overexpression resulted in increased resistance of the LLC-PK1 cells to tacrolimus, cyclosporine A, sirolimus and everolimus [19]. Polymorphisms in the *ABCB1* gene have been found to be associated with a susceptibility to ulcerative colitis [20,21,22,23,24,25], Parkinson’s disease [26,27,28], Alzheimer’s disease [29,30], cancer [31,32,33,34,35], bullous pemphigoid [36,37], the osteonecrosis of the femoral head [38,39], major depressive disorder [40,41,42] and ischemic strokes [43]. A meta-analysis demonstrated an association between the *ABCB1* C3435T polymorphic variant and the dose-adjusted concentration of cyclosporine after a kidney transplantation [44]. In a study involving 84 Greek psoriasis patients, *ABCB1* C3435T polymorphism contributed to a lower ABCB1 activity [45].

Though the genetic polymorphism of ABCB1 may affect the disposition to the drug, produce variable drug effects, and may contribute to the disease risk susceptibility, there has been no pharmacogenetic study which analyzed the relationship between *ABCB1* and the effectiveness of a cyclosporine treatment in the Russian population. The present study aimed to analyze the effect of *ABCB1* polymorphisms on the response to cyclosporine therapies in Russian psoriasis patients who have an *ABCB1* genotype status.

## 2. Methods

### 2.1. Patients

A total of 168 Russian psoriasis patients of European descent were recruited for genotyping (Table 1). The age of the psoriasis patients ranged from 28 to 93 (mean ± standard deviation (SD), 66.3 ± 15.0; 65 females and 103 males). The patients were treated in the City dispensary of Dermatology and Venereology of Saint Petersburg, Russia. Each patient was evaluated according to the standard protocol, including a complete history and physical examination. Descriptive characteristics on psoriasis patient’s demographics, comorbidities, and treatment history were collected. All of the patients had the classical pattern of skin lesions (chronic plaque lesions and psoriasis vulgaris) which were confirmed by a dermatologist. The Psoriasis Area and Severity Index (PASI) was applied to assess the disease activity and the effectiveness of the treatment [45]. All of the patients were treated with cyclosporine Neoral^®^ (NEO, Novartis, Basel, Switzerland) oral solution containing 100 mg of cyclosporine/mL twice daily in a dose of 3 mg/kg/day. The patients had not been administered any other drugs during the period of three months. The patients were classified as responders (PASI score > 75%) and non-responders (PASI ≤ 50%) after three months of treatment with cyclosporine. This study was conducted in accordance with the Declaration of Helsinki. Each individual provided written informed consent before being entered into study. The study was approved by the local hospital ethics committee.

### 2.2. DNA Isolation and Genotyping

Peripheral blood samples of all of the subjects were collected in ethylenediamine tetraacetic acid (EDTA) tube and stored at −70 °C. The genomic DNA was extracted from peripheral blood samples using a standard phenol-chloroform extraction method [46]. The DNA samples were quantified twice using the NanoDrop Spectrophotometer (Thermo Scientific, Waltham, MA, USA). The samples of the genomic DNA were stored at −80 °C until they were used. One hundred and sixty-eight patients treated with cyclosporine were genotyped for *ABCB1* polymorphisms using polymerase chain reaction-restriction fragment length polymorphism analysis PCR-RFLP and verified by TaqMan SNP genotyping assays. The *ABCB1* T-129C, G1199A, C1236T, G2677T/A and C3435T SNPs were selected from a previous study [29] and the National Center for Biotechnology Information (NCBI) website (http://www.ensembl.org; www.ncbi.nlm.nih.gov/SNP (accessed on 1 January 2021)). We followed the genotyping protocol described by Vasilopoulos et al. in 2014 [29]. Primer sequences and annealing temperatures used for the analysis of each polymorphism are summarized in Table 2. We analyzed the T-129C, G1199A, C1236T, G2677T/A and C3435T SNPs in the *ABCB1* gene (Table 2).

We followed the genotyping protocol described by Vasilopoulos et al. in 2014 [45]. The primer sequences and annealing temperatures used for the analysis of each polymorphism are summarized in Table 3. Moreover, the SNPs of the *ABCB1* gene were re-analyzed by a Real-Time PCR using TaqMan^®^ probes—C_27487486_10 was used for T-129C, C_15951365_20 was used for G1199A, C_7586662_10 was used for C1236T, C_11711720C_30 and C_11711720D_40 were used for G2677T/A, and C_7586657_20 for C3435T (Applied Biosystems Inc., Waltham, MA, USA) with TaqMan^®^ Genotyping Master Mix, respectively.

### 2.3. Statistical Analysis

The allele, genotype, and genetic models frequencies and Hardy–Weinberg equilibriums were analyzed using the PLINK version 1.90. The differences in the Hardy–Weinberg equilibrium among all of the individuals in the allele and genetic model frequencies between the studied groups were assessed by Chi square (χ^2^) test. The statistical significance threshold was set to 0.05 for all of the tests. The odds ratios (ORs) and 95% confidence intervals (CI) were calculated where a statistically significant difference in the allele and genetic model frequencies was found. The linkage disequilibrium (LD) block between the SNP pairs in the genomic region of the *ABCB1* locus and haplotypes were estimated using Haploview version 4.1 (Daly Lab, Cambridge, MA, USA) [47,48].

## 3. Results

### 3.1. Allele and Genetic Model Association Analysis with Response to Cyclosporine Treatment

Table 1 shows the clinical characteristics of the psoriasis patients who participated in the current study. The patients were classified as responders (PASI score > 75%) and non-responders (PASI ≤ 50%) after three months of treatment with cyclosporine.

All of the SNPs were in the Hardy–Weinberg equilibrium. The results of the *ABCB1* T-129C, G1199A, C1236T, G2677T/A and C3435T SNP association analysis with response to the cyclosporine treatment are summarized in Table 3. The *ABCB1* C1236T, G2677T/A and C3435T SNPs polymorphisms were significantly associated with a negative response to the cyclosporine therapy. The most significant results were found with C3435T SNP and were associated with an unresponsiveness to the cyclosporine therapy in the allele, dominant and recessive models (Table 4).

The allele 3435T in the *ABCB1* gene demonstrated an association with a negative response to the cyclosporine therapy (OR = 2.58 95%, CI = 1.64–4.06) in the Russian population. Namely, the frequency of allele T of the C3435T SNP in the *ABCB1* gene was significantly higher in the nonresponders compared to that in the responders (0.57% vs. 0.34%, respectively; Table 4). The risk of no response to the cyclosporine treatment was 1.6-fold higher in the carriers of the dominant model TT + CT vs. CC compared with the recessive TT vs. CC + CT model carriers (OR = 4.01, OR = 2.50, respectively). Concerning the G2677T/A SNP, because only one psoriasis patient had an A allele, the carriers of that allele were excluded from the analyses. Moreover, the *ABCB1* C1236T and G2677T/A polymorphisms were significantly associated with a negative response to the cyclosporine therapy in the codominant, dominant and recessive models (*p* < 0.005). In addition, the association analysis revealed no statistically significant difference in the allele and genotype frequencies between the responders and nonresponders for the T-129C and G1199A SNPs.

### 3.2. Haplotype Association Analysis with Response to Cyclosporine Treatment

The haplotype specific analyses of the *ABCB1* polymorphisms are presented in Table 5. The haplotypes with a frequency below 1% were excluded from the analyses, thereby improving the statistical power. The linkage disequilibrium (LD) analysis indicated the existence of one haplotype block (formed by the C3435T, G2677T/A and C1236T SNPs) in the chromosome 7 region among the Russian group. Additionally, the haplotype analysis provided one haplotype that was significantly associated with a negative response to the cyclosporine therapy in the Russian population. Namely, the block 1 haplotype TGC frequency was significantly higher in the nonresponders compared to the responders (0.27% vs. 0.11%, respectively; Table 4). In addition, the association analysis revealed no statistically significant difference in the haplotype frequencies between the responders and nonresponders for the haplotypes CTC, CGC, CTT and TTC.

## 4. Discussion

The development of drug resistance decreases the effectiveness of drug treatment and increases the cost of drug development. In addition, the treatment of psoriasis is associated with a considerable economic burden, with the average annual costs per patient being EUR 11928 in Sweden, EUR 8372 in Italy, and EUR 2866–6707 in Germany based on the treatment type [49]. In searching for the discovery in the multidrug resistance phenotype, many studies have been focused on the *MDR1* gene. Taking into account that cyclosporine is frequently used in the treatment of various autoimmune diseases, such as psoriasis, rheumatoid arthritis, myasthenia gravis, systemic lupus erythematosus and diabetes mellitus, the identification of *MDR1* polymorphisms may help us to find the cause of the ineffectiveness of their therapy.

In this study, we identified the SNPs in the *ABCB1* gene as genetic variants with clinically relevant effects on the psoriasis response of cyclosporine. The *ABCB1* C3435T, G2677T/A, and C1236T polymorphisms were significantly associated with a negative response to the cyclosporine therapy in the allele, codominant, dominant and recessive models. The most significant result was obtained with *ABCB1* C3435T SNP that was associated with a negative response to the cyclosporine therapy. Additionally, the haplotype analysis presented that the TGC haplotype was significantly associated with a negative response to the cyclosporine therapy in the Russian population. Our study had some limitations. Firstly, we did not have a replication cohort in the present study, which would have validated our results, and secondly, our study had a limited sample size. Further studies of different populations are required in order to explore the influence of these variants of the *ABCB1* gene on the effectiveness of the cyclosporine treatment in psoriasis patients.

The 3435T allele frequency is known to vary amongst populations with a high prevalence in the Caucasian population [50]. The synonymous C3435T SNP seems to be of the highest biological importance in protein functioning by changing the mRNA stability, performing alternative splicing or by the modification of the translation efficiency [35]. The homozygous TT genotype is associated with more than two-fold lower duodenal *ABCB1* protein expression when it was compared with the CC samples [15].

Numerous pharmacogenetic studies have revealed and confirmed the C3435T locus as a potential genetic marker of the effectiveness of the drug treatment of psoriasis, epilepsy, organ transplantation and cancer [44,45,51,52,53,54,55,56,57,58,59,60,61,62,63,64,65,66,67,68,69,70]. Vasilopoulos et al. found that there were associations of the C3435T SNP with a negative response to cyclosporine in the Greek population [45]. A meta-analysis demonstrated an association between the *ABCB1* C3435T polymorphic variant and the dose-adjusted concentration of cyclosporine after a kidney transplantation [44]. The effect of C3435T polymorphism on the pharmacokinetics of tacrolimus in liver transplantation was confirmed in Caucasian populations [51]. The studies of organ transplant Caucasian patients have reported on the associations of the C3435T polymorphisms with higher calcineurin inhibitors (CNIs: cyclosporine and tacrolimus) concentrations [52,53,54,55], whereas the association investigations in Asian patients indicated that the C3435T genetic variant did not influence the CNI concentrations [56,57,58,59,60]. Adult cardiac transplant studies have demonstrated inconsistent results [61,62]. In study of 170 heart transplant recipients, the *ABCB1* 3435CC genotype was associated with an increased risk of rejection [61]. Taegtmeyer et al. did not find the *ABCB1* 3435CC genotype to be risk factors for the development of acute rejection [62].

Kwan et al. investigated impact of the *ABCB1* C3435T SNP in drug resistance in 746 Han Chinese people, and they showed a significantly higher the TT genotype frequency in the patients with drug-resistant epilepsy [63]. However, the effect the C3435T SNP was not confirmed in some studies that were conducted on Caucasian populations [64,65]. Nevertheless, a meta-analysis in 3,912 drug-resistant epileptic patients and 4,419 epileptic patients established the association C3435T polymorphism with drug resistance in epilepsy in a Caucasian population [66]. A subsequent meta-analysis detected the association of the *ABCB1* 3435TT genotype with the risk of antiepileptic drugs resistance [67].

Numerous associations’ studies and meta-analyses have established the impact of the *ABCB1* rs1045642 genetic variant in cancer treatment outcomes concerning chemotoxicity, overall survival, and therapeutic responses. The impact of the C3435T SNP on the imatinib response in chronic myeloid leukemia (CML) patients have been widely estimated, however, the results of the studies were contradictory. A meta-analysis by combining data from a total of 12 studies including 1826 patients indicated that the 3435T allele predicted a worse response to imatinib in CML patients. Loscocco et al. found that the *ABCB1* 3435TT genotype correlated with a higher probability to achieve an MR3 molecular response in a shorter time in 90 CML patients that were treated with nilotinib [68]. Gregers et al. observed higher liver toxicity values after high-dose methotrexate was administered to acute lymphoblastic leukemia patients with the 3435CC variant versus the 3435CT/TT one [69]. In a meta-analysis involving nine (770 patients), five (566 patients) and three studies (367 patients), no significant association of C3435T in a dominant genetic model with a response to chemotherapy in patients with breast cancer was seen [70]. However, the results did not change after the stratification by ethnicity, cancer type and response criteria [70]. Schaich et al. examined the association of the genetic variants of the *ABCB1* gene with the survival of glioblastoma patients who were treated with temozolomide in [71]. The *ABCB1* 3435CC genotype is associated with a 37% survival rate in glioblastoma patients after a temozolomide treatment [71]. The carriers of the homozygous 3435CC genotype had a higher survival rate when they were compared with the heterozygous genotype carriers in an Indian population [72]. Malmström et al. found a significant correlation with survival for the *ABCB1* G1199A SNP in a Swedish population, with the median OS for the homozygous GG patients being 18.2 months versus 11.5 months for the heterozygous AG (*p* = 0.012) [73].

In total, there are inconsistent results in the studies regarding the effects of *ABCB1* polymorphisms on the treatment response in patients with different diseases, and these can be due to them having small sample sizes, inter-ethical variability, differences in concomitant medication and using single time-point sampling [74].

## 5. Conclusions

In conclusion, we demonstrated a strong association between the cyclosporine therapy in psoriasis and the *ABCB1* C1236T, G2677T/A and C3435T SNPs. Further studies with a larger sample size are needed to verify this issue. The detection of pharmacogenetic markers of a treatment response may be useful in predicting the clinical response to psoriasis therapies, and these would help in the improvement of personalized therapy. In future, combination OMICs technologies with known clinical and immunological data will allow us to identify the potential pharmacogenetic markers and therapeutic targets [3]. 

## Figures and Tables

**Table 1:** Demographic and clinical information.

Subphenotype	Sample Set
Number of cases	168
Male: female ratio of affected	103:65
Age affected at entry to the study	
Mean ± S.D.	66.3 ± 15.0
Range	28–93
Number of affected with age of onset	
<40 years (type I psoriasis)	135
>40 years (type II psoriasis)	33
PASI at baseline, mean ± S.D.	14.75 ± 3.77
PASI at 3 months, mean ± S.D.	4.72 ± 3.10
Responders, percentage	104 (63%)
Nonresponders, percentage	64 (37%)

S.D.—mean ± standard deviation. PASI—The Psoriasis Area and Severity Index.

**Table 2:** *ABCB* single nucleotide polymorphisms (SNPs) analyzed in this study.

No	SNPs	Position	Location	Variant	Mutation	Amino Acid Change
1	rs3213619	chr7:87600877	Exon 1	T-129C	5’-UTR	
2	rs2229109	chr7:87550493	Exon11	G1199A	missense	Ser400Asn
3	rs1128503	chr7:87550285	Exon 12	C1236T	synonymous	Gly412Gly
4	rs2032582	chr7:87531302	Exon 21	G2677T/A	missense	Ala893Ser/Thr
5	rs1045642	chr7:87509329	Exon 26	C3435T	synonymous	Ile1145Ile

**Table 3:** List of SNPs, primer sequences, type of enzymes used for PCR-RFLP analysis in this study.

SNPs	Primers Sequence	Annealing Temperature (°C)	Restriction Enzyme, Incubation Conditions
T-129C (rs321361)	F 5′-ATTGGCTGGGCAGGAACA-3′ R 5′-TTTGGAAGATACTCCGAC-3′	58 °C	*Msp* II, 37 °C,16 h
G1199A (rs222910)	F 5′-CAGCTATTCGAAGAGTGGGC-3′ R 5′-CCGTGAGAAAAAAACTTCAAGG-3′	57 °C	*Eco*57I, 65 °C, 20 min
C1236T (rs112850)	F 5′-TCTTTGTCACTTTATCCAGC-3′ R 5′-TCTCACCATCCCCTCTGT-3′	58 °C	*Hae*III, 37 °C, 4 h
G2677T/A (rs203258)	F 5′-TGCAGGCTATAGGTTCCAGG-3′ R 5′-GTTTGACTCACCTTCCCAG-3′	58 °C	*Bsr*I, 65 °C, 4 h
	F 5′-TGCAGGCTATAGGTTCCAGG-3′ R 5′-TTTAGTTTGACTCACCTTCCCG-3′	58 °C	*Ban*I, 37 °C, 4 h
C3435T (rs104564)	F 5′-TAGGCCAGAGAGGCTGCC-3′ R 5′-AGTGGCTCCGAGCACACC-3′	58 °C	*Mbo*I, 37 °C, 4 h

F: Forward primer. R: reverse primer.

**Table 4:** Association analysis of SNPs from *ABCB1* gene with response to cyclosporine therapy at 3 months.

SNP	Genetic Model	Nonresponders PASI ˂ 50 ^a^ p No *n* = 64	Responders PASI ˃ 75 p No *n* = 104	^b^ χ^2^	^c^ *p*-Value
C3435T (rs1045642)	Allele model T vs. C	74/54	72/136	17.35	**3.1 × 10^−5^**
	Codominant model TT vs. CT vs. CC	22/30/12	18/36/50	15.6	**0.0004**
	Dominant model TT + CT vs. CC	52/12	54/50	14.6	**0.00013**
	Recessive model TT vs. CC + CT	22/42	18/86	5.45	**0.02**
G2677T/A (rs2032582)	Allele model T(A) vs. G	50/78	98/110	2.08	0.148
	Codominant model T(A)/T(A) vs. GT(A) vs. GG	8/34/22	30/38/36	7.2	**0.027**
	Dominant model T(A)/T(A) + GT(A) vs. GG	42/22	68/36	0.001	0.974
	Recessive model T(A)/T(A) vs. GG + GT(A)	8/56	30/74	6.04	**0.013**
C1236T (rs1128503)	Allele model T vs. C	48/88	80/128	1.79	0.180
	Codominant model TT vs. CT vs. CC	8/24/32	12/56/36	4.57	0.101
	Dominant model TT + CT vs. CC	32/32	68/36	3.89	**0.048**
	Recessive model TT vs. CC + CT	8/56	12/92	0.03	0.851
G1199A (rs2229109)	Allele model T vs. C	8/120	24/184	2.57	0.108
	Codominant model TT vs. CT vs. CC	0/8/56	0/24/80	2.57	0.885
	Dominant model TT + CT vs. CC	8/56	24/80	0.04	0.871
	Recessive model TT vs. CC + CT	0/64	0/104	3.03	0.552
T-129C (rs3213619)	Allele model T vs. C	4/124	10/198	0.56	0.453
	Codominant model TT vs. CT vs. CC	0/4/60	2/6/96	0.48	0.735
	Dominant model TT + CT vs. CC	4/60	8/96	0.37	0.599
	Recessive model TT vs. CC + CT	0/64	2/104	0.78	0.532

Significant results are shown in bold face. ^a^ p No: genetic model frequency, ^b^ χ^2^: chi-square, ^c^ *p*: *p*-value.

**Table 5:** Haplotype association analysis of SNPs from *MDR1* gene with response to cyclosporine therapy at 3 months.

Haplotype	Nonresponders PASI < 50 ^a^ p No (%) *n* = 64	Responders PASI > 75 p No (%) *n* = 104	^b^ χ^2^ Statistic	^c^ *p*-Value
^d^ Block1 (chromosome 7 region in a Russian population is formed by C3435T, G2677T/A, C1236T SNPs				
TGC	0.27	0.11	13.34	**2.0 × 10^−4^**
CTC	0.11	0.18	2.86	0.090
CGC	0.15	0.22	2.23	0.135
CTT	0.07	0.14	3.65	0.055
TTC	0.14	0.09	1.98	0.158

Significant results are shown in bold face. ^a^ p No: genetic model frequency, ^b^ χ^2^: chi-square, ^c^ *p*: *p*-value, ^d^ haplotype combinations with less than 1% frequency are not displayed.

## Data Availability

The data that support the findings of this study are available from the corresponding author upon reasonable request.
